# Towards routine, city-scale accessibility metrics: Graph theoretic interpretations of pedestrian access using personalized pedestrian network analysis

**DOI:** 10.1371/journal.pone.0248399

**Published:** 2021-03-19

**Authors:** Nicholas Bolten, Anat Caspi

**Affiliations:** Paul G. Allen School of Computer Science and Engineering, University of Washington, Seattle, Washington, United States of America; Univ. Lyon, ENTPE, Univ. Gustave Eiffel, FRANCE

## Abstract

A wide range of analytical methods applied to urban systems address the modeling of pedestrian behavior. These include methods for multimodal trip service areas, access to businesses and public services, diverse metrics of “walkability”, and the interpretation of location data. Infrastructure performance metrics in particular are an increasingly important means by which to understand and provide services to an urbanizing population. In contrast to traditional one-size-fits all analyses of street networks, as more detailed pedestrian-specific transportation network data becomes available, the opportunity arises to model the pedestrian network in terms of individual experiences. Here, we present a formalized and city-scale framework, personalized pedestrian network analysis (PPNA), for embedding and retrieving pedestrian experiences. PPNA enables evaluation of new, detailed, and open pedestrian transportation network data using a quantitative parameterization of a pedestrian’s preferences and requirements, producing one or more weighted network(s) that provide a basis for posing varied urban pedestrian experience research questions, with four approaches provided as examples. We introduce normalized sidewalk reach (NSR), a walkshed-based metric of individual pedestrian access to the sidewalk network, and sidewalk reach quotient (SRQ), an estimate of inequity based on comparing the normalized sidewalk reach values for different pedestrian profiles at the same location. Next, we investigate a higher-order and combinatorial research question that enumerates pedestrian network-based amenity access between pedestrians. Finally, we present city-scale betweenness centrality calculations between unique pedestrian experiences, highlighting disagreement between pedestrians on the “importance” of various pedestrian network corridors. Taken together, this framework and examples represent a significant emerging opportunity to promote the embedding of more explicit and inclusive hypotheses of pedestrian experience into research on urban pedestrian accessibility, multimodal transportation modeling, urban network analysis, and a broader range of research questions.

## Introduction

With technology’s advanced ability to track mobility and sense environmental factors, evidence is accumulating that individuals do not always follow the shortest path, whether in private vehicles [1–4] or using other modalities [5]. Scientists are taking steps to identify conditions influencing human mobility, from understanding route choices made due to travel urgency and purpose (e.g., regular workday routines vs. serendipitous or circumstantial travel) to capturing travelers’ heterogeneous preferences, wants and requirements about path attributes, e.g. both static characteristic of paths (like stairs being inaccessible to travelers with strollers, wheelchairs or scooters) and transient attributes (like rain or crowds or lighting conditions making certain paths undesirable at certain times). It is therefore widely understood that many interrelated factors influence individuals’ route choice.

In a diverse array of applications such as optimization of transportation systems [6, 7], path flow estimation or spread of infectious disease [8–10], route choices made by individuals are summed and aggregated. A large body of work found significant predictability in the *aggregated patterns* of human mobility, primarily in the form of scaling properties and power laws; these suggest that certain stochastic processes can be used as model proxies of aggregated human mobility patterns [11–18]. However, many questions that arise in modern urban and transportation planning applications, particularly those regarding individual route planning, urban accessibility or transportation equity, require analysis from a particular subpopulation or individual’s perspective on the transportation network. In these cases, it is imperative to articulate aspects of personal human mobility, needs, abilities and preferences in order to properly account for the personal cost of traversal over every edge and node in the transportation graph when modeling transportation or route choice.

Modeling pedestrian travel modes presents a particular challenge, then, given heterogeneity in personal abilities, travel purpose, and preferences. Such heterogeneity creates a wide spectrum of personal costs to utilizing any edge, node or subgraph of the overall pedestrian transportation network. Pedestrian-related challenges faced by urban and transportation planners are often treated with a myopic focus on a single urban problem, or transit service, or sidewalk issue, rarely addressing this variability. For example, traditional methods for studying neighborhood spatial accessibility (measuring the ease with which residents can access community amenities) display aggregation error effects and severe biases because they ignore the travel network altogether (“as-the-crow-flies” distance calculations), assuming pedestrians mobilize only in the road network, or aggregating entire neighborhoods or blocks into single points of origin [19, 20]. Despite well-established studies that explore pedestrians’ route choice priorities and the many factors (mentioned above) affecting route preferences, very few studies or methods apply any criteria other than minimizing travel time or distance in models to forecast individual choices at large scales of urban networks. While a primary issue here concerns the availability of data for expressing the actual pedestrian transportation graph (to distinguish from the automobile road graph), another issue arises from the general lack of information on how each factor contributes to individual choice and to what extent.

Network-based pedestrian access analyses are dominated by the use of a street network as the primary data layer and model pedestrians as lacking individual constraints when traversing network elements. Such approaches overlook known diversity in pedestrian experiences, e.g.,, disability populations, but are often the only practical and scalable means by which to evaluate pedestrian access due to a dearth of (1) well-annotated pedestrian transportation networks and (2) a representative enumeration of pedestrian navigational requirements. Consequently, research into pedestrian access has focused on enumerating and visualizing a handful of infrastructure elements at city-scale [21], small-scale (neighborhood or smaller) evaluations that consider pedestrian diversity [22–24], or large-scale network models based on a monolithic description of the pedestrian experience [25]. However, emerging means to scalably collect detailed pedestrian network data, including crowdsourcing in OpenStreetMap [26] and computer vision approaches [27–31] suggest that large-scale pedestrian networks will become increasingly available resources for investigating complex urban research questions. In addition, the detailed modeling of pedestrian behaviors and needs is increasingly suitable for scientific test and evaluation through the use of location tracking technology [32–35] and detailed surveys [36, 37].

The confluence of detailed pedestrian network data and characterizations of pedestrian preferences provide the opportunity to include pedestrian needs and considerations that have otherwise been historically overlooked. Omnipresent constraints on pedestrian mobility, such as those reported by wheelchair, walker, or white cane users, can be reformulated as a set of profiles of pedestrian mobility by which to evaluate a given pedestrian network, improving inclusivity in existing analyses and potentially framing new avenues of investigation. Conditional constraints, like those that arise when pushing a stroller, are amenable to the same paradigm.

The need to incorporate a diversity of concerns, interpreting a single network according to a wide set of PMPs, as well as efficiency in collecting data, calls for the publication of pedestrian networks that, rather than embedding subjective interpretations a priori, are characterized by an increasingly large set of physical or legal metadata. For example, some pedestrian network mapping approaches embed evaluations of wheelchair accessibility directly in network elements [38], wherein accessibility for all wheelchair users is subjectively prejudged by a third party. However, wheelchair users exhibit significant diversity in their navigational preferences, using chairs of varying widths, describing differing preferences on steepness, and recalling differing propensities to attempt conditional barriers like raised curbs; therefore, their experiences cannot be encapsulated by a single binary evaluation. A network appropriate for the navigational variation among wheelchair users (as well as all pedestrians) should instead embed path width, steepness, and curbs metadata that can be interpreted in downstream analysis.

Here we propose the use of pedestrian mobility profiles (PMPs) where an individual (or a subpopulation) might be represented as a vector of cost parameters determining composite internal costs of travel through particular environments. The profiles are used as a parametric expression of factors and weights of factors that impact route choice and traversal through pedestrian paths. This work presents a general framework, Personalized Pedestrian Network Analysis (PPNA), as a mechanism of integrating pedestrian mobility profiles into route forecasting over large-scale pedestrian networks. We demonstrate several network analyses in which pedestrian mobility profiles can significantly alter optimal routes and even graph properties (e.g., in the case where a pedestrian mobility profile drives edge costs to render an edge non-traversable to that person or subpopulation). Additionally, by exploiting the variation among pedestrians and PMPs, we can examine pedestrian accessibility questions at large-scale via comparative analyses of graphs and subgraphs that result when different pedestrian mobility profile traversals are superimposed on the same urban pedestrian graph. For example, we can compare the graph with costs evaluated by imposing the pedestrian mobility constraints of an individual with mobility limitations to the graph evaluated by imposing a PMP with no constraints other than preference for shortest travel time in order to identify subgraphs of the urban network. These comparative analyses help us answer, in a consistent and equitable manner, questions about neighborhood spatial accessibility or access to transportation that have hitherto, in real-world applications, been conducted using survey or GPS data at limited sample sizes and travel range.

This work is organized as follows. After addressing materials and methods, we introduce the main concepts of Personalized Pedestrian Network Analysis (PPNA) by presenting pedestrian mobility profiles and how network evaluations arise from calculating the costs of traversing graph components conditioned on a pedestrian mobility profile. We then illustrate the wide applicability, nuanced analytic contributions, and diversity-enhancing properties of the PPNA approach with a series of graph-analytic examples that highlight (1) interrogating pedestrian network walksheds, (2) equity in access to pedestrian spaces, (3) equity in access to public amenities and services, and (4) the identification of central network corridors that are distinct between individual pedestrians.

## Materials and methods

Pedestrian and street network data were retrieved from the OpenSidewalks Project [39], which publishes open transportation network datasets based on agency and OpenStreetMap data. Network modeling and manipulations were carried out using the Entwiner and Unweaver Python packages [40, 41], with the networkx Python library used for some algorithms (betweenness, shortest-paths) [42]. Visualizations of pedestrian network analytics metrics were generated using either the GeoPandas or plotnine Python libraries and QGIS. Pedestrian preference data were gathered using both formal surveys and informal interviews of pedestrians (in prior work). All code and data necessary to replicate the analyses and figures in this paper are available at the OpenSidewalks GitHub organization.

Three pedestrian profiles, individualized network traversal parameterizations of the pedestrian experience, are used throughout this study as exemplars: a normative walking profile, a manual wheelchair profile, and a powered wheelchair profile. All three are stereotyped and do not adequately capture the diversity in pedestrian experience and requirements within each group, and this paper will precede specific profiles with the word “stereotyped”. The stereotyped normative walking profile is parameterized as having no restrictions when traversing the pedestrian network and can also be considered the more typical representation of pedestrian movement within the literature: a pedestrian who moves at constant speed and can use all sidewalks and crossing locations. The stereotyped manual wheelchair user represents an individual who requires lowered curbs when crossing the street and who cannot ascend inclines greater than 8% or descend slopes greater than 10%. The stereotyped powered wheelchair user represents an individual who requires lowered curbs when crossing the street and who cannot ascend inclines greater than 10% or descend slopes greater than 12%. These correspond roughly to pedestrian preferences reported in previous informal interviews of pedestrians who use these devices and to Americans with Disabilities Act (ADA) guidelines on access ramps. Again, each profile is a known stereotype; for instance, some manual wheelchair users indicated no concerns about climbing much steeper inclines than most powered wheelchair users and indicated a willingness to traverse any raised curb.

## Results

### Personalized pedestrian network analysis

**Fig 1:**
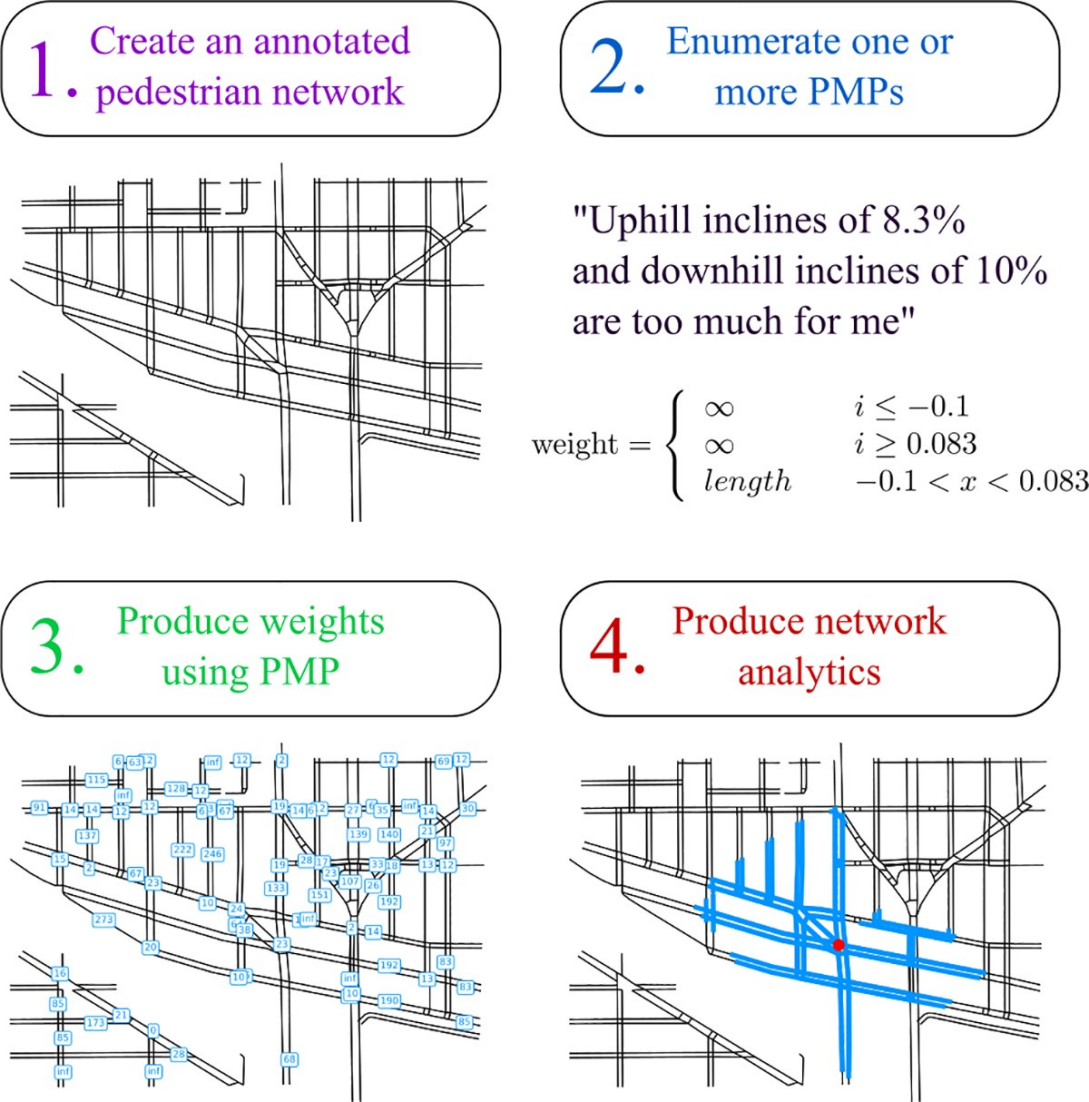
Step-by-step personalized pedestrian network analysis. (1) An annotated pedestrian network is generated from a geospatial dataset. In this case, a network of sidewalks and street crossing locations annotated with path length and incline (grade or slope) information was interpreted as a graph where edges are sidewalks and crossings, and nodes are where these elements meet end-to-end. (2) One or more pedestrian mobility profiles (PMPs) are enumerated, representing an individual pedestrian who may or may not represent a larger population. Individual pedestrian preferences are translated into a quantitative pedestrian mobility profile, which parameterizes a cost function that returns a numerical weight (infinity or a real number) based on metadata stored in a network edge (e.g., a sidewalk). (3) This PMP-parameterized cost function is evaluated over every edge in the network. This panel shows 1/15th of all weights to avoid overplotting. (4) Any appropriate or exploratory spatial network-based analysis can now be applied to this individually-weighted network. This panel shows the set of reachable paths for a stereotyped manual wheelchair user starting at one point in the Fremont neighborhood of Seattle, WA, where reachability is defined as a filled-in shortest-path tree.

We first describe a general-purpose framework for routinely bridging informational gaps in pedestrian access: personalized pedestrian network analysis (PPNA, Fig 1). PPNA simulates link-level pedestrian experiences by evaluating each element of the pedestrian network as if it were being traversed by one particular pedestrian with specifically-stated needs and requirements (a PMP) that are interpreted as parameters to a cost function, producing a single numeric value or network edge weight. This framework promotes the simultaneous use of multiple pedestrian mobility profiles (PMPs) to embed multiple weights (and therefore pedestrian experiences) into a particular graph-analytic investigation. While each element used in the PPNA approach has precedent in previous work, including crowd-contributed pedestrian network graphs [43], agent-based models [24], and evaluations of a detailed pedestrian network [44], the combination of these methods has not been explored as a routine means by which to understand and evaluate pedestrian access for wider applications. When a city-scale pedestrian network is evaluated using a specific PMP, it is transformed into a map of pedestrian experiences that is amenable to a wide range of graph-analytic paradigms and suitable for incorporation into the regular practices of city planners, disability advocates, and policymakers.

Transportation network data, including a pedestrian network, consist of graph structures derived from spatial relationships. In personalized pedestrian network analysis (PPNA), an edge represents a segment of a pedestrian path, and a node represents a potential “decision point,” i.e., a point where pedestrian paths meet (i.e., a node). If two nodes are described as u and v, respectively, an edge connecting them can be described as (u, v), typically a traversable path segment in spatial networks. Path metadata can be stored along with the barebones graph description of an edge, represented as the triple (u, v, d), with d being any set of associated metadata such as steepness, path width, or surface composition. Because traversal will frequently vary depending on the direction of travel (uphill vs. downhill, up or down stairs, up or down a curb), personalized pedestrian network analysis reinterprets a detailed asset-level description of a pedestrian network in which there is typically a single data object representing an edge and then derives a directed multigraph. A multigraph, which can have multiple edges with the same start and end nodes, is favored over a graph in order to realistically account for branching and joining of alternative pedestrian paths. For example, a wheelchair ramp and a steep path may each be represented by a single edge that begins and ends at the same location. In this case, there exist two directed edges with the same abstract graphical representation, (u, v).

Therefore, the most appropriate pedestrian network derivation for PPNA is a directed multigraph comprised of nodes connected by (u, v, d) edge triples. A PMP operates on these edge triples to derive a numerical weight of traversal, or impedance, and stores this number uniquely within the metadata d. For example, a pedestrian who has difficulty with hills may be partially described by a PMP cost function that accepts edge length and incline as inputs and yields an estimate of the time-of-travel, embedded in the edge metadata, d. Because PMPs are necessarily discrete and explicit in terms of the demographic concern(s) they describe, their routine incorporation via PPNA may serve to raise the profile of specific type(s) of pedestrians represented in a given analysis; potential limitations may be more easily recognized; and intentional oversights may be enumerated. For example, evaluating a detailed pedestrian network for regulatory compliance (such as compliance with the Americans with Disabilities Act) may be explicitly formulated as a highly-constrained PMP that rejects any network elements that present out-of-compliance barriers or infrastructure by assigning an infinite edge weight. This PMP would not necessarily represent any living pedestrian, but instead it would represent a theoretical floor of access that explicitly prioritizes a wide set of pedestrians. Under such a formulation, it becomes immediately clear that other concerns have been diminished: a pedestrian using crutches may actually prefer a (e.g., faster) route other than one identified as ADA compliant, but this preference has not been accounted for. Similarly, a PMP focused exclusively on physical barriers will lend itself to access questions purely related to traversability but will necessarily lack insights into concerns that extend beyond this factor, like safety, time-of-travel, considerations of pleasantness, or fatigue. Because pedestrian needs and preferences are currently not well-enumerated, a PMP-based analysis framework emphasizes the specification of a particular research approach and who is considered.

A network generated using PPNA can be analyzed in isolation or combined with higher-order, pedestrian-centric research questions. In isolation, a network interpreted through PPNA may reveal spatial or topological insights into the relationship between diverse pedestrians and their environment through direct analysis of the underlying graph structure. For example, PMP evaluation for a stereotyped manual wheelchair user who requires lowered curbs will reveal disconnected subgraphs wherever a neighborhood has poor curb ramp coverage since the PMP will generate high or infinite weights for street crossings that lack lowered curbs. These street crossing weights can be spatially summarized and compared to other metrics of potential relevance for an area of interest (AOI) to create an intersectional analysis of pedestrian access. For example, an AOI with poor link-level connectivity for a certain PMP may also coincide with demographic or economic inequalities, such as food deserts, implying greater spatial inequalities of outcome than would otherwise be recognized. A weighted graph with poor link-level connectivity for a given PMP is a network structure and can therefore also be used to pose or constrain any common graph-based research questions. For example, a research question that probes ease of pedestrian movement between different zones of activity, such as commercial-residential connectivity, can be reframed as targeted shortest-path traversals from all points of interest in one zone to all points of interest in another.

These examples and considerations have elaborated on the use of PPNA as a general-purpose approach to pedestrian network modeling that can enrich existing analyses and promote a pluralistic paradigm for studying pedestrian access. The following sections highlight the application of PPNA to common questions in pedestrian access analysis and the derivation of deeper insights through city-scale interpretations of diverse pedestrian experiences.

### Probing pedestrian access to the sidewalk network

There are various interpretations or definitions of pedestrian access, with many addressing whether a given pedestrian can reach destinations of interest, potentially subject to some constraint. In this paper, we frame pedestrian access as the ability to reach or traverse elements of a pedestrian network subject to the constraint of one or more pedestrian mobility profiles (PMPs). For illustrative purposes, we emphasize use cases where pedestrians state specific mobility requirements or related mobility impairments since such concerns have been frequently overlooked in mainstream applications. Nevertheless, all of the following examples could instead be organized around PMPs evaluating other concerns like perceived trip pleasantness, fastest routes for runners given varying inclines and surfaces, expected crowdedness, and/or noise levels.

#### Walksheds

**Fig 2:**
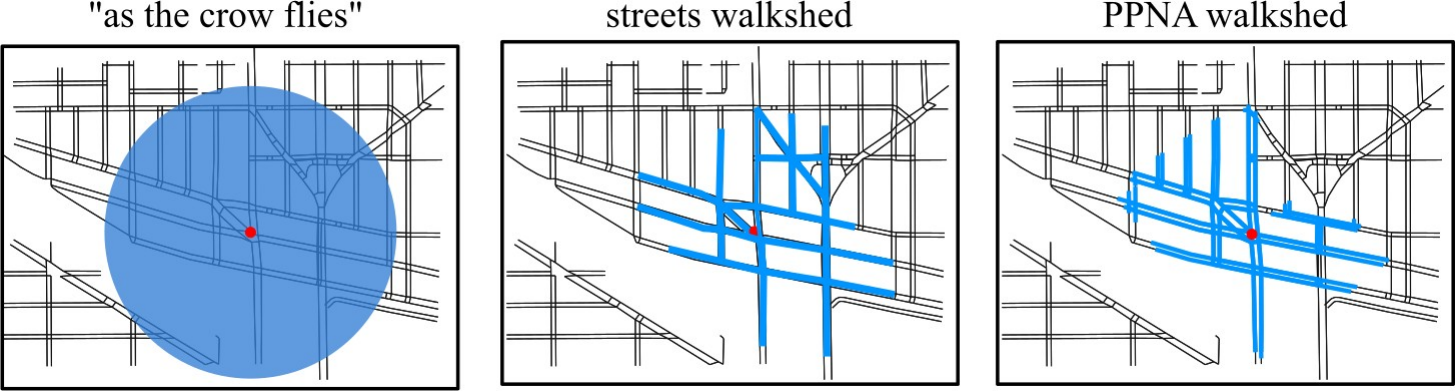
Alternative technical formulations of 400-meter pedestrian service areas. (Left panel) A circular buffer, calculated “as the crow flies” from a point of interest, is overlaid on the transportation network without concern for physical barriers. (Center panel) A street-based walkshed that uses a monolithic pedestrian model more realistically models traversal of the street network but overlooks pedestrian-specific infrastructure and navigational concerns. (Right panel) A PPNA-based walkshed reveals areas inaccessible to a stereotyped manual wheelchair user. An area in the Northeast section of this map was considered reachable by the street walkshed approach but not by the PPNA approach due to steep sidewalks between the Northwest “Y” intersection and the starting point.

A common research question in pedestrian access, particularly for transit agencies, is how to estimate a service area from which a particular asset or point of interest can be reached by pedestrians. For example, a transit agency may have a mandate to evaluate the areas, demographics, and individuals served by a particular bus line, represented as the areas reachable from one or more bus stops. However, the strategies by which a service area has been calculated vary significantly in their appropriateness and specificity to pedestrian travel. One strategy is to assume travel “as the crow flies,” or a circular buffer, in which a perfectly circular service area is generated around a point of interest (Fig 2, left panel). This approach, though straightforward to calculate and therefore widely deployed, is fundamentally inaccurate since it assumes there exist no physical or legal barriers for any direction of travel; a circular buffer service area necessarily overestimates pedestrian access. While the limitations of circular buffer areas have been known for many years and have fallen by the wayside in literature focused on physically modeling pedestrian movement, they still frequently appear in academic and agency literature to explicitly or implicitly describe pedestrian access or walkability [45–53].

Another way to estimate a pedestrian service area is to produce a network-based estimation of pedestrian path reachability, also known as a walkshed, pedshed, or metric reach [54] (Fig 2, center panel). A walkshed estimates a service area by simulating traversal of a transportation network; however, it has traditionally been limited to street networks that are interpreted using a monolithic and simplistic model of pedestrian behavior, usually considering only distance of travel, such as a quarter mile (roughly 400 meters), or a constant speed of travel regardless of environmental conditions. By relying on the street network, street-based walksheds avoid errors inherent to an “as-the-crow-flies” method, e.g., direct pedestrian travel through buildings, walls, waterways, or other inaccessible land uses. Instead, street-based walksheds model pedestrians as traveling along the centerlines of certain classes of streets (high-speed raised motorways are generally excluded from analysis). In addition, some primarily street-based walksheds, such as those derived from Google Maps™, include a handful of alternative pedestrian paths such as those extending through parks or large public stairways, creating a hybrid pedestrian network.

Though a significant improvement over “as-the-crow-flies” service areas, such street-based walksheds nevertheless suffer from inherent inaccuracies: pedestrians rarely travel down the center of streets, it is often unsafe or illegal to attempt to do so, and real pedestrians possess a complex set of pedestrian mobility needs and preferences regarding dedicated pedestrian infrastructure that are not represented. In addition, the use of street-based walksheds does not necessarily produce better results than a circular buffer for some analyses [53, 55], which may indicate street network analysis is missing important information that may be captured by more detailed pedestrian networks, such as paths through buildings, desire lines, sidewalk information, and street crossing information. This gap separating seemingly more detailed network modeling from applied predictive work may also be due in part to the lack of a coherent framework to help practitioners avoid inappropriate approximations of pedestrian access models.

#### Applying personalized pedestrian network analysis to walksheds

Walksheds can be made more realistic and inclusive by imposing personalized pedestrian network analysis (PPNA). Rather than relying solely on a street network, a PPNA walkshed accounts for dedicated pedestrian paths such as sidewalks and street crossings and interprets network traversals via one or more pedestrian mobility profiles (PMPs) (Fig 2, right panel). Beginning at a point on or near the network, a PPNA walkshed simulates a specific pedestrian’s experience as a directed shortest-path tree traversal of a detailed pedestrian network, halting either when a maximum metric has been reached or when a hard barrier blocks further travel along an edge. By default, a shortest-path tree enumerates only nodes reached within the cutoff distance. Therefore, producing a complete walkshed requires extending the fringe of the tree to include partial edges, conditioned on the PMP of interest (Fig 1, right panel showing partially-traversed edges).

#### A pedestrian infrastructure-centric walkability metric: Normalized sidewalk reach

Many iterations of the concept of “walkability” attempt to estimate pedestrians’ ability to reach a variety of amenities and other services on foot by aggregating diverse metrics like street-based walksheds, the density of sidewalks, and the density of amenities or zones of distinct economic activity (commercial, residential, public services). WalkScore, targeted at real estate applications, famously publishes a map of “walkability” pixels, summarizing walkability as a single numeric value that applies to a large (several thousand square meter) area. While WalkScore and similar aggregated metrics of walkability have demonstrated some correlations with perceived walkability, associated pedestrian behaviors, or safety [56], they have been shown to create aggregation error and biases [19, 57]. Moreover, they are, intuitively, limited in appropriateness and specificity due to their lack of detailed pedestrian networks or diverse pedestrian preferences.

**Fig 3:**
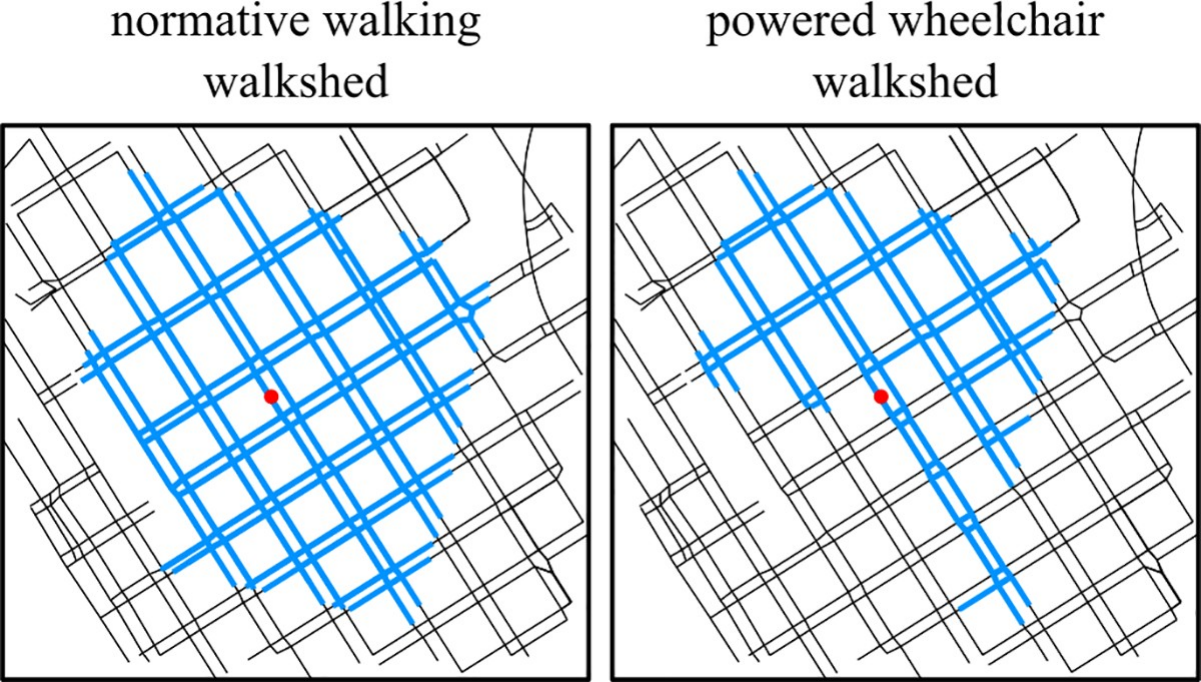
Distinct PMPs generate distinct walksheds. Each panel shows a PPNA-based walkshed originating at the same location but modeled with a different PMP. (Left) A normative walking PMP walkshed, reaching a wide, roughly diamond-shaped area in downtown Seattle. (Right) The walkshed of a more-constrained PMP that stereotypes a powered wheelchair user with moderate incline constraints and a requirement for lowered curbs when crossing the street. It is approximately ⅔ the size of the normative PMP walkshed, missing a large segment on the Southwest area due to steep hills and the lack of lowered curbs in the downtown neighborhood.

The ability to evaluate a personalized pedestrian network analysis (PPNA)-based walkshed for any point near the pedestrian network represents a data-motivated path forward for evaluating one component of walkability: sum link-level traversability of a given space. Fig 3 shows PPNA walksheds produced by different PMPs at the same location, indicating distinct pedestrian experiences from the same point of exploration. The visual differences between these walksheds suggests an inequality in access: to the pedestrian network, but how might access to the pedestrian network be quantified in the first place? One straightforward way to compare pedestrian access is to treat the network itself as a desirable destination for pedestrians: total network distance reachable by a given pedestrian subject to a maximum distance (or time) constraint.

Total walkshed distance suffers from spatial and infrastructure biases, however. Compare walkshed sum distances with a single PMP for different urban spaces. One space may have a larger sum distance due solely to density of roads and infrastructure, but have less than 50% sidewalk coverage, while another space may have a low sum walkshed distance but full coverage due to having only a handful of roads. A metric of sidewalk access should be invariant to density or configuration of the street network. This can be framed as a need to normalize by an alternative hypothesis: what is the ideal expectation of sidewalk coverage given known street infrastructure?

**Fig 4:**
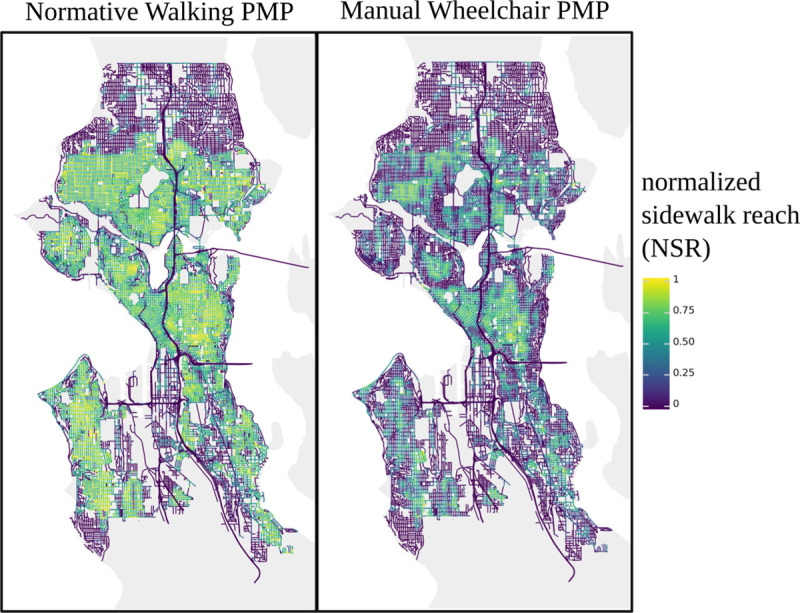
Normalized sidewalk reach for normative and manual wheelchair PMPs for every street in Seattle, WA. Normalized sidewalk reach was evaluated at the center point of every street in Seattle using either a normative (left) or stereotyped manual wheelchair (right) PMP. These normalized sidewalk reach values reveal granular spatial variation in access as well as a visual means by which to compare city-scale pedestrian accessibility between two pedestrians. Namely, the normative PMP generated NSR values exceed 0.75 for three large, contiguous regions representing the North Seattle, Central Seattle, and West Seattle/Delridge areas, whereas the wheelchair PMP generated NSR values rarely exceed 0.75, with many small islands of relative accessibility divided by large regions of poor (less than 0.25) NSR values. The figure contains information from OpenStreetMap and OpenStreetMap Foundation, which is made available under the Open Database License.

Accounting for these challenges, we introduce a normalized, infrastructure-specific metric of sidewalk network accessibility, *normalized sidewalk reach* (Fig 4). Normalized sidewalk reach is a unitless metric proportional to the quotient of two walksheds: the division of the metric reach of a specific PMP-evaluated pedestrian network walkshed by that of an optimistic PMP-evaluated street walkshed. The denominator quantity, or normalization factor, can therefore be considered an approximation of the optimistic null hypothesis where all streets have sidewalk infrastructure; normalized sidewalk reach can also be thought of as an estimate of the fraction of space reachable on existing sidewalk infrastructure versus a scenario in which all streets have fully accessible sidewalks. We formally define normalized sidewalk reach in Eq 1, where *l* refers to the length of a path segment within a walkshed, with a suffix (*ped* or *street*) distinguishing the network for which a walkshed was evaluated. The numerator is the sum total of segment lengths reachable on the pedestrian network (subject to a specific PMP), and the denominator is the sum total of segment lengths reachable on the street network according to a permissive (normative) pedestrian profile. Normalized sidewalk reach therefore approximates the actual pedestrian network distance reachable relative to an ideal case alternative hypothesis of full coverage of accessible sidewalks and crossings. A regularization term of ½ is introduced to account for the expectation that each street could have a maximum of two sidewalks. A given normalized sidewalk reach value represents the relative ability of a particular pedestrian (represented by a PMP) to access a pedestrian network similarly to an idealized, pedestrian-accessible street network. A value of 0 would indicate a street without sidewalks, a value near 1 would indicate similar accessibility between the sidewalk and street networks, and an intermediate value would indicate partial (and proportional) accessibility of the sidewalk network.

$$Normalized\;sidewalk\;reach(P_{i})=f(P_{i})=\frac{\sum l_{ped_{P_{i}}}}{\sum l_{street_{P_{normative}}}}$$ (1)

**Eq 1. The definition of normalized sidewalk reach**.

A normalized sidewalk reach value neither represents nor attempts to represent a general-purpose metric of “walkability”. Instead, it serves as an alternative metric of access to the sidewalk network that could be incorporated into a downstream walkability analysis. The development of normalized sidewalk reach is a direct consequence of applying and interpreting the PPNA approach to understanding pedestrian networks, entirely contained within the methodology.

### Probing equity questions of pedestrian sidewalk access

Equity in pedestrian access is of primary importance for regulatory, urban analytic, and advocacy concerns. For many research questions, the difference in outcome between classes or groupings of pedestrians is of primary interest, not just the raw access metrics for a single definition of the pedestrian experience. For example, city planners are often tasked with prioritizing equitable outcomes for infrastructure investment that weigh not just the state of infrastructure for an area of interest, but equity concerns regarding demographics. Such planning decisions must also often weigh the expected cost of development since complex funding models may make improving infrastructure in areas with the lowest need and equity concerns cheaper. Rather than relying on human practices to manage such complexity, the introduction of metrics of pedestrian access equity could assist in such decision-making processes.

In Fig 4, each panel visualizes the normalized sidewalk reach (NSR) value for every street in the city of Seattle, Washington for a single PMP, with color corresponding to the NSR. Under the assumption that equity is focused on equality in outcome between populations, ideally holding all other variables constant, access to the same infrastructure between pedestrian populations, as represented by PMPs, inherently raises questions of equity. In this section, we present an illustrative, personalized pedestrian network analysis (PPNA)-based metric of equity, *sidewalk reach quotient*, that compares pedestrian network walksheds between pedestrian mobility profiles.

**Fig 5:**
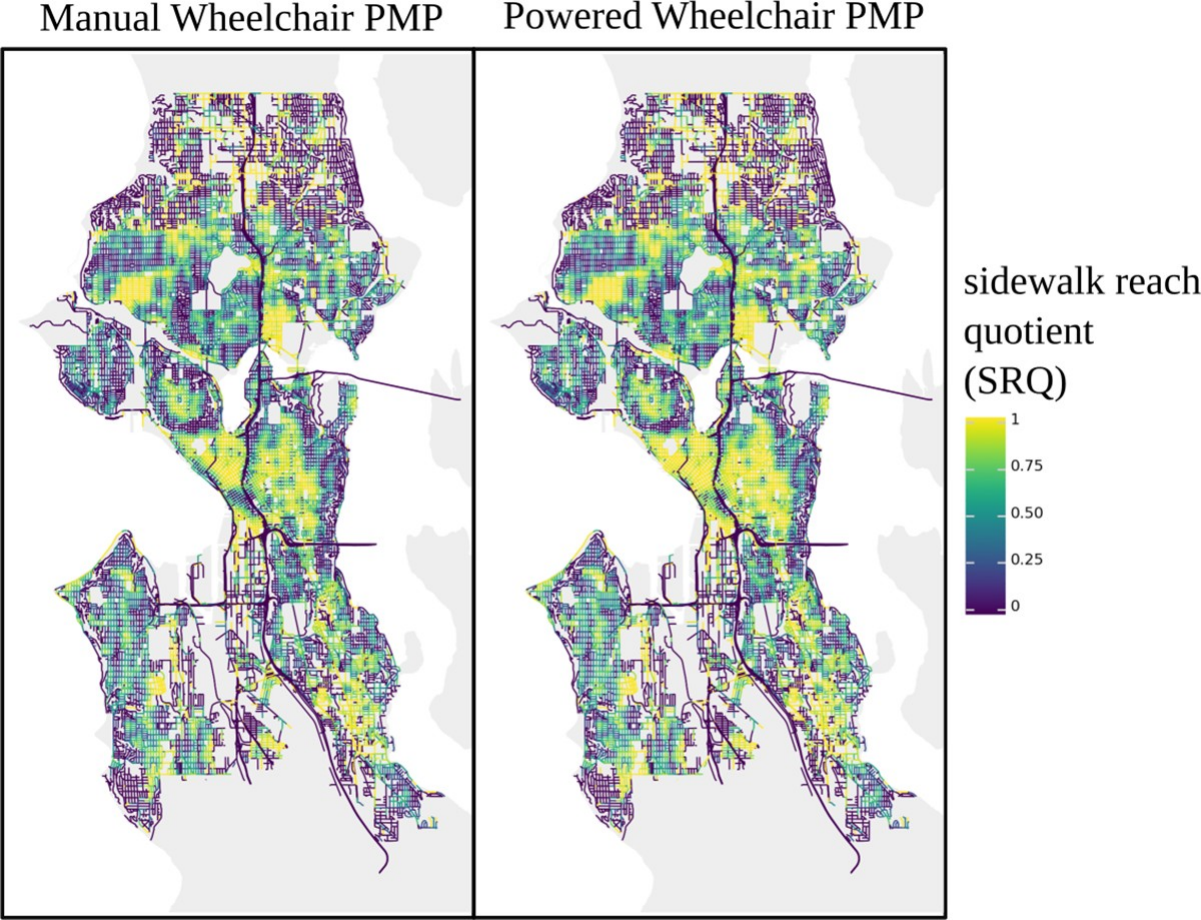
Sidewalk reach quotients for manual and powered wheelchair pedestrian mobility profiles for every street in Seattle, WA. Sidewalk reach quotients provide a (relative) quantitative basis of comparison for access to public sidewalk infrastructure between two pedestrians profiles. They were evaluated at the center point of every street in Seattle using either a stereotyped manual wheelchair (left) or stereotyped powered wheelchair (right) pedestrian mobility profile (PMP) for the numerator and a normative walking PMP for the denominator. The stereotyped powered wheelchair PMP is less constrained than the stereotyped manual wheelchair PMP since powered wheelchair users tend to report fewer concerns about steep inclines. While the maps produced by both PMPs exhibit a “splotchy” pattern, indicating wide spatial variation in this equity metric, the stereotyped manual wheelchair PMP frequently produces lower SRQ values. For example, the downtown region (near central, Western coast) has noticeably higher frequencies of high-SRQ values for the stereotyped powered wheelchair profile than for the stereotyped manual wheelchair one. This figure contains information from OpenStreetMap and OpenStreetMap Foundation, which is made available under the Open Database License.

Given a quantitative metric based on a single pedestrian mobility profile, a derivative personalized pedestrian network analysis equity metric can be defined as any numerically valued function that compares values of this metric based on at least two different mobility profiles. Two obvious equity metrics would be the absolute difference between access metrics and the quotient of one metric divided by another, creating a factor of comparison. An access difference metric in this context would always be framed in units of the pedestrian access metric, corresponding to distance in the case of a walkshed metric or unitless for normalized sidewalk reach. Because a numerical difference value must be carefully interpreted in terms of the units of the associated access metric, sidewalk reach quotient (SRQ) is a factor-based metric of pedestrian equity. Specifically, sidewalk reach quotient is defined as the numerical value produced by dividing the normalized sidewalk reach for one PMP by that of a different PMP, holding the on-network starting position constant. Unless otherwise noted, PMP used for the denominator is a normative (least-constrained) pedestrian mobility profile (Fig 5 and Eq 2). A low sidewalk reach quotient indicates that the PMP of interest generated a much smaller walkshed than the normative profile, suggesting a significant inequity; a sidewalk reach quotient approaching 1 indicates a similarly sized walkshed, or relative equity between the PMPs examined. Because the denominator is identical for both normalized sidewalk reach values in the sidewalk reach quotient equation, this quantity cancels out during division and the sidewalk reach quotient can be calculated directly from pedestrian walksheds without calculating a street walkshed. Therefore, the sidewalk reach quotient can be considered the relative sidewalk walkshed reach of one PMP versus another for a particular location in space, comparing bulk distances of reachable paths.

$$sidewalk\;reach\;quotient(P_{i})=\frac{NSR{(P}_{i})}{NSR(P_{normative})}$$ (2)

**Eq** 2**. The formulation of sidewalk reach quotient.**

As demonstrated in Fig 5, equity in access to the sidewalk network is highly spatially variable in the city of interest, with contiguous regions, or islands, of equitable access divided by large areas of inequitable access. In addition, differences in experience between stereotyped wheelchair populations can be identified between the panels of Fig 5, with downtown Seattle (central, near Western coast) showing higher levels of pedestrian access equity for a stereotyped powered wheelchair user than for a stereotyped manual wheelchair user. PMP-based pedestrian access metrics can reveal city-scale inequities across arbitrary populations since they provide a common base of comparison: the pedestrian network itself.

### Assessing diversity in pedestrian access to amenities

**Fig 6:**
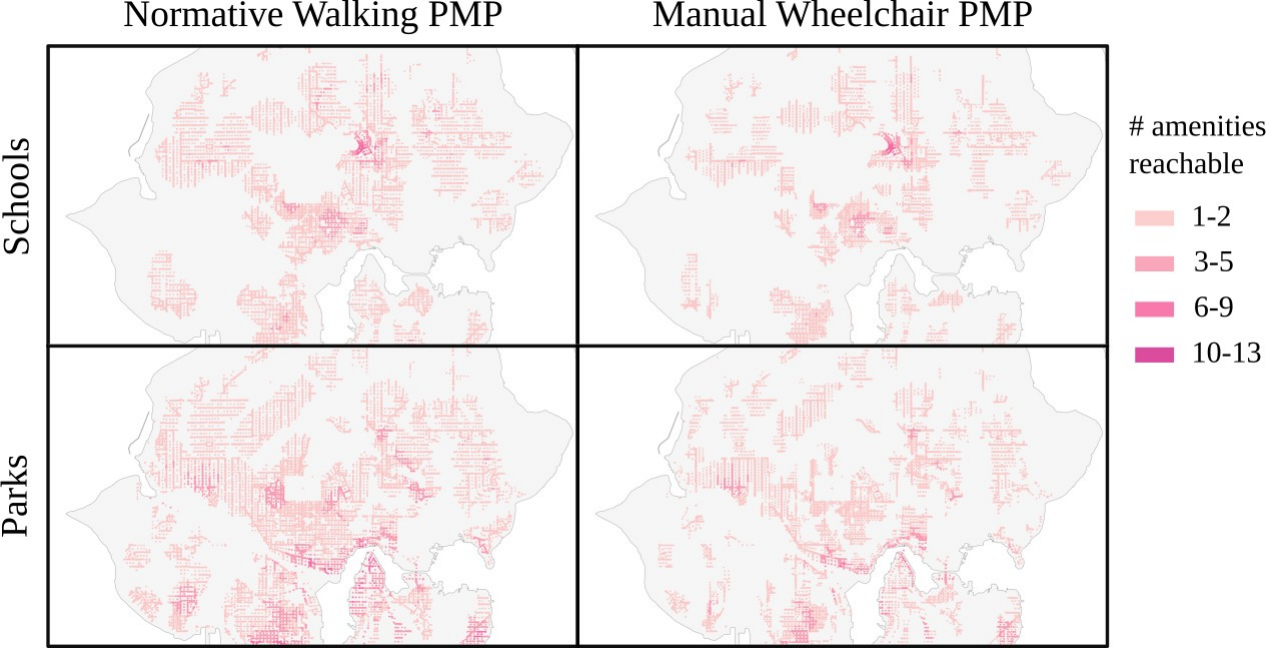
Per-sidewalk amenity access for normative and manual wheelchair PMPs for each street in the Seattle, WA region. Access to two amenity categories (parks and schools, vertical axis) were evaluated by 400-meter walksheds using two pedestrian mobility profiles (stereotyped normative walking and stereotyped manual wheelchair, horizontal axis). For both amenity categories, the manual wheelchair PMP is constrained by inclines and the use of lowered curbs, leading to a distinctly smaller set of service areas. In both cases, the overall number of streets that can reach at least one example of the amenity (a school or public park) is dramatically smaller for the stereotyped manual wheelchair profile. In addition, the quantity reachable is noticeably higher (darker coloration) for the normative profile for both amenities. This figure contains information from OpenStreetMap and OpenStreetMap Foundation, which is made available under the Open Database License.

In earlier sections, walksheds (via normalized sidewalk reach) were used to evaluate access to pedestrian infrastructure, producing a quantitative means to evaluate pedestrian sidewalk access without concern for reaching or embarking from a particular amenity or activity. Applying the PPNA framework to questions of access to amenities, more typical pedestrian access questions can be evaluated at scale while representing the diversity of pedestrian needs. Fig 6 compares pedestrian access to two amenities (schools and parks) in the Seattle, Washington area based on 400-meter walksheds, summing the number of unique locations within a given amenity class that can be reached starting at a point on the sidewalk network. Two pedestrian mobility profiles are considered, one for a stereotyped manual wheelchair user with strong constraints on inclines and lowered curbs, and another for a stereotyped powered wheelchair user with weaker constraints on inclines. Owing to frequent hilly neighborhoods, stark differences in access are apparent between the two pedestrian models, with about ⅓ fewer sidewalks reaching at least one school or park for the stereotyped manual wheelchair profile versus the stereotyped powered wheelchair profile.

In addition to the expected inequities in access to public amenities, this analysis provides a means by which to quantify absolute access and service levels by neighborhood and district. Thus, by incorporating PPNA into amenity access, researchers can determine differential access to services.

### Pedestrian network centrality

Many network-based research questions attempt to quantify the importance of specific network (graph) elements such as graph connectivity, identifying the highest-degree node, and centrality, i.e., how frequently a given network node or edge appears under a given traversal consideration. For example, street network centrality has been used to drive research questions concerning the impact of transportation network topology on economic activity [58].

It is not feasible to enumerate all means by which personalized pedestrian network analysis (PPNA) can be applied to these graph analytic approaches. Instead, this section presents illustrative examples of outcomes produced by applying PPNA to common metrics of link-level edge importance. First, we identify graph connectivity as a simple yet revealing analysis on urban spaces, demonstrating the extent to which an urban space becomes disconnected into impassable (for pedestrians) islands based on PMPs. Then, we evaluate betweenness centrality at city-scale for varying PMPs, identifying the best-connected infrastructure for traversal by different populations.

### Personalized pedestrian network analysis-modified graph connectivity

**Fig 7:**
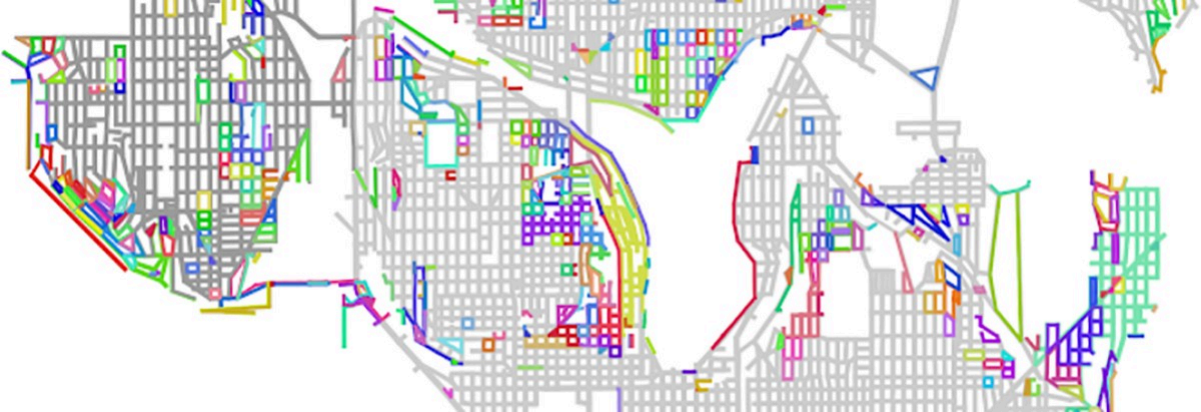
Disconnected subgraphs in the pedestrian network. A map shows sidewalks that have been uniquely colored based on sharing a unique subgraph. Subgraphs result from disconnections in the street crossing network as interpreted by a stereotyped manual wheelchair PMP; a stereotyped manual wheelchair user would have difficulty entering or leaving these subgraphs as a pedestrian due to the lack of lowered curbs. Over 100 disconnected subgraphs are shown, with one neighborhood (Madison Park) thoroughly and multiply disconnected from the larger pedestrian network.

A fundamental property of any graph is connectivity, i.e., whether all components are connected or whether there are disconnected subgraphs. The pedestrian transportation network gathered for the city of Seattle is a connected graph by default, but when evaluated by a pedestrian mobility profile that requires lowered curbs to cross the street, it rapidly splinters into a large number of pedestrian access “islands” (Fig 7). Without the imposition of a specific pedestrian experience, this level of discontinuity would not be discovered, and a wide range of pedestrian concerns would not be accounted for. Pedestrians with stability concerns, pedestrians using wheeled assistive mobility devices, and pedestrians with temporary injuries, such as those using knee scooters, would experience this urban space in a drastically different way than would-be predictions made using the normative pedestrian model. Therefore, for large segments of a given city, self-guided exploration imposes an unreasonable burden due to these hard barriers and trip planning services are likely necessary for reliable and expedient navigation.

#### Betweenness centrality under different pedestrian concerns

**Fig 8:**
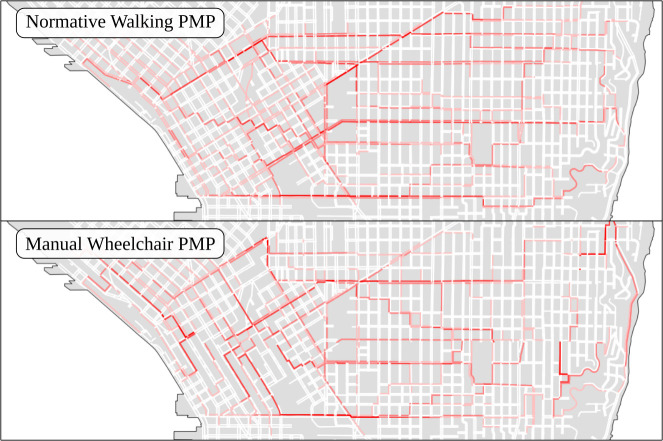
400-meter betweenness centrality for normative walking and manual wheelchair PMPs in Seattle, WA. Betweenness centrality evaluated for a central, rectangular region of Seattle, WA. Values are normalized and unitless but share a color map, with darker colors corresponding to more central (higher betweenness) network segments. The upper panel shows betweenness centrality for sidewalk network segments evaluated by a stereotyped normative walking pedestrian mobility profile while the lower panel shows the same for a stereotyped manual wheelchair PMP. While some contiguous sets of pedestrian network elements have high betweenness within both profiles, indicating consensus on betweenness between the profiles, several disagreements become apparent. The downtown region (Western/left section of the map) has conspicuously divergent paths of high-betweenness values. Its normative profile produced more evenly distributed betweenness and a single major path in contrast to the manual wheelchair profile, which produced a small and different set of high-betweenness paths with very low betweenness values surrounding them, reflecting the steepness of downtown Seattle in the Southwest/Northeast directions. This figure contains information from OpenStreetMap and OpenStreetMap Foundation, which is made available under the Open Database License.

A graph analytic approach to identifying potentially important or otherwise explanatory network elements is betweenness centrality: given an all-shortest-paths analysis of graph nodes to all other graph nodes (potentially subject to a traversal cutoff weight), edge betweenness centrality counts the number of shortest paths that use a particular network edge (e.g., a sidewalk). In this framing, the units compared to determine what a “shortest” path entails are conditioned on the cost function used to evaluate each network edge and can represent units of time, distance, or a more complex combination of concerns. Fig 8 shows betweenness centrality for 400-meter shortest paths in Seattle, Washington evaluated over different stereotyped pedestrian PMPs.

Alternative PMPs produce starkly different patterns in centrality, suggesting infrastructure that may be of vastly differential utility depending on pedestrian mobility concerns. City planners or permitting authorities will not recognize such potential bottlenecks or important corridors without quantitative approaches that compare mobility or other pedestrian concerns. For example, a highly central corridor of the pedestrian transportation network for stereotyped wheelchair users in the Southeast corner of the area of interest running South to North is not present for stereotyped normative pedestrians. As a path connecting residential zones to a major school and a commercial district, it may be the only direct path available for wheelchair users to access these amenities, and any disruption to the sidewalk network (construction, parade routes, natural disasters) may produce a highly inequitable outcome that would otherwise be overlooked.

## Discussion

In the preceding sections, we presented illustrative cases addressing the utility of imposing parameterizations of the pedestrian experience on a detailed, city-scale pedestrian network for downstream analysis. In some cases, this approach was developed in order to create direct scoring metrics of accessibility (normalized sidewalk reach, sidewalk reach quotient), or used to constrain common network metrics (walksheds, graph connectivity, centrality), demonstrating amenability to being routinely incorporated into urban analysis. In all scenarios, the quantitative introduction of pedestrian diversity into the analysis provided deeper insight by promoting a more detailed modeling of pedestrian concerns, highlighting exactly which pedestrian concerns were being considered (and which were not) and promoting the use of a more realistic pedestrian network from which to derive such insights.

Because personalized pedestrian network analysis framework has such broad potential application for any question regarding pedestrian access, many analyses that we did not present here could be of value in future work. Linking distinct economic zones is of primary interest to transit agencies, and while neither transit stops nor their ability to connect pedestrian spaces were evaluated in this study, these research questions would be enriched by a PPNA approach. Vision Zero and other pedestrian safety efforts could be well served by more nuanced models of pedestrian behavior and would likely benefit from more varied network metadata than is currently available. There is a panoply of pedestrian concerns that are rarely evaluated but of potentially greater importance that would be appropriate for a pedestrian mobility profile and therefore a personalized pedestrian network-based analysis. The pleasantness of a pedestrian trip may factor in greenways, availability of covered walkways to avoid sun, rain, or snow and quantify them in terms of a PMP. The amenability of a space for safe and legal recreational use by wheeled sports equipment may quantify a PMP with similar infrastructural preferences for wheelchair populations, but with entirely different parameterizations. Individuals with respiratory concerns may benefit from a PMP that prioritizes indoor routes, routes known to have high-quality air purification, or those that avoid vehicular traffic. In addition, the PPNA approach could be adapted to evaluate the completeness or appropriateness of both real and planned pedestrian infrastructure projects, such as attempted implementations of the Complete Streets specification.

All pedestrian access metrics explored in this work are a priori estimations of pedestrian access concerns and can be interpreted as hypotheses about potential real-world pedestrian experiences. These hypotheses should be tested against real-world data using methods like surveys, location tracking and map matching, and ethnographic practices. Aside from testing the potential validity of these pedestrian models, real-world data can drive refinement and improvement of how pedestrian concerns are modeled and how likely trade-offs are weighted. For example, in informal interviews, pedestrians with a variety of mobility concerns have communicated a willingness to attempt traversal of barriers they previously identified as impassable in order to save substantial amounts of time. Real-world pedestrian behavior will therefore certainly vary from a priori expectations and suggest what those trade-offs may be, providing an opportunity to approximate the diversity of pedestrian experience from behavioral data in a PPNA approach.

The pedestrian network presented here, while at much larger scale and specificity than those previously explored, remains limited. The problem of the last 50 meters of travel remains, with the trip from primary connecting pedestrian infrastructure to a realistic destination being unspecified. When assessing access to amenities, no datasets specifying the exact entrances to those facilities or the intervening pedestrian infrastructure were available, forcing guesswork and estimations that contradict some of the core premises of a PPNA-based approach: the traversability of pedestrian infrastructure is of primary importance for evaluating access. Indoor spaces are increasingly important for urban spaces and traversable pathways, both to access amenities and as an intermediate route between destinations but are largely unmapped. Furthermore, because large-scale, detailed mapping of pedestrian spaces is an emerging phenomenon [59, 60], many classes of pedestrian infrastructure are not mapped or have poorly defined primitives like plazas (not well-described as a path with a width), street crossings (most relevant street furniture is unmapped), or the absence of physical landmarks for blind navigation.

There is significant potential for advocacy work in elevating the importance of a diversity of pedestrian experiences. Some of the work presented in previous sections (walksheds) was successfully used to advocate for providing access for disability populations to public buildings after-hours in downtown Seattle since indoor elevators connected important transit hubs but were otherwise unavailable. Prior to a data-driven formulation, these concerns could be communicated only anecdotally by individuals. However, as a human-contingent analysis of infrastructure at scale, they became vital data on which civic action was premised.
